# Psychophysiological effects of a web-based stress management system: A prospective, randomized controlled intervention study of IT and media workers [ISRCTN54254861]

**DOI:** 10.1186/1471-2458-5-78

**Published:** 2005-07-25

**Authors:** Dan Hasson, Ulla Maria Anderberg, Töres Theorell, Bengt B Arnetz

**Affiliations:** 1 Uppsala University, Department of Public Health and Caring Sciences, Section for Social Medicine/CEOS, Uppsala Science Park, SE-751 85 Uppsala, Sweden; 2 IPM – The National Swedish Institute for Psychosocial Medicine, Granits väg 8, SE-171 77 Stockholm, Sweden

## Abstract

**Background:**

The aim of the present study was to assess possible effects on mental and physical well-being and stress-related biological markers of a web-based health promotion tool.

**Methods:**

A randomized, prospectively controlled study was conducted with before and after measurements, involving 303 employees (187 men and 116 women, age 23–64) from four information technology and two media companies. Half of the participants were offered web-based health promotion and stress management training (intervention) lasting for six months. All other participants constituted the reference group. Different biological markers were measured to detect possible physiological changes.

**Results:**

After six months the intervention group had improved statistically significantly compared to the reference group on ratings of ability to manage stress, sleep quality, mental energy, concentration ability and social support. The anabolic hormone dehydroepiandosterone sulphate (DHEA-S) decreased significantly in the reference group as compared to unchanged levels in the intervention group. Neuropeptide Y (NPY) increased significantly in the intervention group compared to the reference group. Chromogranin A (CgA) decreased significantly in the intervention group as compared to the reference group. Tumour necrosis factor α (TNFα) decreased significantly in the reference group compared to the intervention group. Logistic regression analysis revealed that group (intervention vs. reference) remained a significant factor in five out of nine predictive models.

**Conclusion:**

The results indicate that an automatic web-based system might have short-term beneficial physiological and psychological effects and thus might be an opportunity in counteracting some clinically relevant and common stress and health issues of today.

## Background

Stress-related disorders are major public health issues in many industrialized countries and are expected to become increasingly common in the coming decades [1,2]. Such disorders have a negative economic impact, disrupt work and home life and might even increase suicide risk [3]. Numerous web-based health sites and tools are being offered to the public for stress management and treatment of stress-related mental conditions such as depression and anxiety. More and more people are rapidly using these sites and tools. The majority of the "health seekers" rely on search engines and seldom check the source and date of online health information [4,5]. According to Fox & Fallows (2003), about 93 million Americans (half of American adults) have searched online for health information. Few prospectively controlled intervention studies have been published on the efficacy of these health sites and web-based tools. Even fewer of them have assessed both psychological and physiological effects. There are increasing indications, however, that Internet-based intervention programs have beneficial effects on various psychological conditions and other desired outcomes [6-8]. Moreover, prospectively controlled studies without physiological evaluation have indicated beneficial effects from computer- or web-based tools on, for example, headache [9], distress related to tinnitus [10], depression and anxiety [11-13], stress management [14], physical activity [15] and insomnia [16].

Considering these indications from previous studies, web-based interventions for stress management and health promotion may offer promising opportunities. Some possible advantages with web-based interventions compared to more traditional alternatives, such as books, coaches and therapists may be the 24-hour accessibility, possibility for interaction, instant feedback and support. Moreover, the scalability and potential reach of web-based interventions may further be an advantage in economical terms for individuals, corporations and the society. One major disadvantage and risk, however, is the lack of quality assurance of web-based health sites and interventions. There are no international agreed upon guidelines for assessment, and users of these services may receive misleading or incorrect information that may potentially be harmful to health and wellbeing [17].

The present study was conducted during a stressful period for the IT and media companies in Sweden. For the information technology companies, there was downsizing after the dot-com bubble burst. The media companies worked intensively in covering the election campaign to the Swedish parliament. The study population, however, had been chosen to be representative for a future that employees will face more and more frequently: increasing pace of changes, shorter status quo time, "project work" and other challenges that were new to knowledge workers. This implies that the results from the present study may be applicable more generally to employees in the future: How they will react in a stressful situation that directly affects the basic conditions of one's organization and workplace.

A multitude of biological and physiological markers have proved to be related to stress, health and recovery. Some markers have been thoroughly investigated in various studies, whereas there is limited information on others. Moreover, relationships between biological markers and stress, health and recovery seem to be complex since many factors may affect their patterns of secretion, including negative feedback and secretion of other related hormones [18]. For some hormones, such as cortisol, immune markers and sex hormones, it is also essential to consider seasonal changes and natural variation in daily cycles [19]. Clearly, more knowledge is needed regarding longitudinal relationships between biological markers, stress, health and recovery. Most of the biological and physiological markers to be analyzed in the present study have been assessed in relation to short- and long-term stress in previous studies. Some of the markers, such as blood status, were to be routinely sampled for overall health matters or general profiling that could indicate alterations in plasma volume.

The aim of the present study was to assess the possible effects on mental and physical well-being and biological stress markers from a web-based stress management and health promotion tool. It was expected that decreased levels in indicators of catabolism and increased levels in indicators of anabolism would be found in the intervention group compared to the reference group at the end of the study. To our knowledge, this is the first completely web-based assessment, where self-ratings are complemented with biologically relevant outcome data.

The web-based system was developed and adjusted with the aim of making it useful as a tool for everyday life, including usage on a daily or regular basis. Therefore it had to be easy to use, time-efficient and accessible through the work place and at home 24 hours a day. The participants were recruited at worksites since the study included some organizational aspects, such as health economics, not presented in this paper.

## Methods

### Participants and study groups

**Figure 1:**
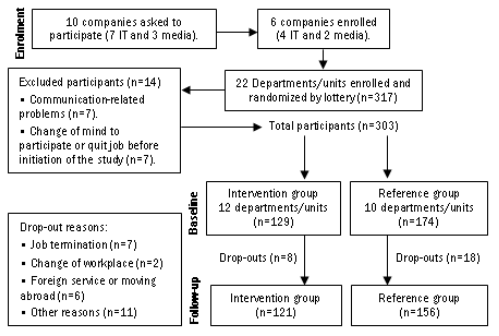
**Flow of participants. **The figure illustrates the flow of participants through each stage of the randomized trial. Additionally, the numbers of excluded participants and drop-out rates, including generalized reasons for these actions are depicted.

Flow of participants throughout the study is depicted in Figure 1. In collaboration with a White-Collar Union (Sif) and a Swedish Employers' Association (Almega), ten companies insured by the study's source of funding Alecta (an occupational pension plan company) were asked as to their interest in participating. The asked companies were selected and contacted by employees at Alecta, by mail and phone. The management departments of six out of the ten asked companies were interested. Informed of the basic inclusion criteria, i.e. minimum group of ten individuals and access to economic production data, 2–4 departments within each company were chosen and asked by the company management as to their interest in participating. The managers of the selected departments in turn asked their employees whether they were interested in participating. No incentives were offered to the participants, with exception of the extensive blood sampling including feedback of the results, which seemed to be a motivator for many participants.

For some companies the departments were located in different cities and for some in the same buildings or city. With the exception for one of the media companies where a whole department with five units enrolled, there was no "natural" connection between the participating departments. Consequently, there was only occasional risk of contamination of the extended intervention to the reference group. Moreover, the design of the websites for the intervention and reference group respectively was similar in appearance (see more information on the interventions below). The intervention group website only had two additional buttons, which makes it hard to notice any difference if the site would be exposed to a participant of the reference group.

The participating departments were, within each company, randomized by lottery to either the intervention or reference group. Thus each company had at least one intervention and one reference group. All departments received a 30-minute information session including 10 minutes for questions and answers. These information sessions included the aim of and general information about the study as well as general information on stress and health. Finally, after oral information each participant received written information about the project and consent forms. All the participants were informed, orally as well as in writing, that participation was voluntary and withdrawal was possible at any time.

There is no information on the exact number of employees that were asked to participate in the study. An exception was one of the media companies where 95 out of 100 possible participants chose to participate. In general there was also a great interest from the other departments and similar participation rates is therefore estimated. Altogether, 317 participants from 22 departments/units in four information technology and two media companies enrolled in the study. Fourteen participants were excluded because of communication-related problems (n = 7), change of mind to participate or quit their job before initiation of the study (n = 7). Thus 303 persons finally participated in the study, out of which 26 participants (8.6%) dropped out. The reasons for dropping out were job termination (n = 7), change of workplace (n = 2), foreign service or moving abroad (n = 6) or other reasons (n = 11). There were no significant differences in dropout rates between the groups (6.9% in the intervention group vs. 9.8% in the reference group, p between groups = n.s.). Nor were there any significant differences between the intervention and reference groups in socioeconomic background or psychophysiological measures at baseline. Regarding the participants, there was no information about possible mental or somatic disorders or medication.

The participants had professions such as IT technicians, programmers, system developers as well as journalists/reporters, news presenters, sound technicians and photographers. The main type of work-site was open plan offices. Many participants from the IT-companies were partly located in the work sites of their customers for longer or shorter periods. For the media companies, some participants, such as photographers and reporters, were partially ambulatory and worked in different locations. The common feature for all participants was regular and daily computer usage at work.

### The web-based tool

Table 1 provides a detailed description of the web-based tool and illustrates similarities and differences in the features that were offered to the intervention and reference group respectively. A web-based tool for health promotion and stress management was developed and offered all participants real-time monitoring of perceived current health and stress status, a diary and information about stress and health (Table 1). In addition, participants in the intervention group were offered web-based cognitive exercises, aimed at decreasing unwanted stress and promoting health and recovery through health promotion initiatives. The exercises included techniques for relaxation, time management, cognitive reframing and a chat. Thus, the only things that distinguished the groups were the addition of the cognitive exercises and the chat in the intervention group. The web-based tool was developed by the researchers and most techniques are commonly utilized techniques in cognitive and behavioral psychology and stress management. These techniques were modified so that they could become more or less self-instructing to be used for self-help purposes.

**Table 1:** The web-based tools. The table depicts the different features included in the web-based tool for the study groups respectively. The only things that distinguished the groups were the addition of the cognitive exercises and the chat in the intervention group.

**Feature**	**Intervention group**	**Reference group**
*Monitoring tool for stress and health levels with instant feedback; graphs illustrating current and retrospective ratings and an option to compare results with other groups with the same socioeconomic profile, within the same department/company and all the respondents in the data base. The questionnaire was compiled by a ten-item questionnaire for regular or daily usage*.	YES	YES
*Diary connected to the monitoring tool so that ratings and notes could be compared and examined retrospectively. The diary could be used as stress management but also as a tool for improving self-knowledge and how different events affect health and well-being*.	YES	YES
*Popular scientific information on stress and health compiled by various Swedish researchers*.	YES	YES
*Self-help in the form of classical stress management exercises for; relaxation and sleep improvement, cognitive reframing, time-management, emotional control and self-knowledge, strengthening self-esteem, life reflection, dissociation*.	YES	**NO**
*Chat*	YES	**NO**

The web-based tool and the exercises were not pilot tested before the study. However, the tool as well as exercises were chosen and adjusted on the basis that they had to be time efficient in order to be utilized. It was hypothesized that basic demands for regular usage were instant feedback on the questionnaire and that the measurement and exercises could be used rapidly. Consequently, it was decided that regular or daily monitoring should not take more than 20–40 seconds. Moreover, every exercise was labeled with information of time for accomplishment (time span 1–60 minutes). Some of the cognitive exercises, e.g. improving self-confidence, were designed such that they consumed 5–10 minutes when learning and then could be conducted in a matter of seconds when utilizing.

Most exercises were presented in three different modes; on the web-site as plain text, as a downloadable PDF-file (sometimes including descriptive images), and as a flash animation, guiding the participant with image and sound through the exercise. Since the intervention for both groups was completely web-based it could only be accessed online. All information was however printable, which made it possible for the participants to print material of interest and thus intervene elsewhere. Exposure to the intervention for both groups could only be logged via the number of logins to the website.

### Questionnaire

A questionnaire was compiled and included about 100 questions concerning socioeconomic status, consumption of caffeine drinks, expectations about the research project, self-rated health (SRH), stress and wellbeing at work as well as during leisure time, health economics and performance at work (Table 2). Most of the questions were presented as Visual Analogue Scales (VAS) and some, concerning health economy, work time, basic daily functioning and symptoms of ill health, were presented as multiple-choice questions. Most of the newly constructed single VAS questions were based on previously validated Likert-based items or indices [20-25]. Participants filled out the questionnaire online at baseline (before the initiation of the study) and at the end of the six-month intervention.

**Table 2:** Questionnaire. The table illustrates theoretical models, items and topics covered by the questionnaire. Most items were presented as "straight forward" VAS, e.g. How is your overall sleep quality (Very poor – Very good).

**Models**	**Topics – generalized self-ratings**
*Socioeconomic and background factors*	Age, sex, annual income and self-rated financial situation, educational level, marital status, possession of children, work role (co-worker, middle-manager, manager), amount of customer contact, duration of current working position, smoking habits, satisfaction with eating habits, consumption of coffee, tea, soft drinks and energy drinks. Expectations of the possible effects of the research project on stress and health level.
*Lifestyle, health promoting and compromising behaviours, cognitive function, sense of coherence and wellbeing*	Self-rated health (last year, right now and future expectations), sleep quality, memory, concentration ability, ache in various body parts, physical exercise habits, mental energy, frequency and source (home, work or combination) of stress, stress management ability, satisfaction with leisure-time, life goals, communication ability with others, meaningful life, future optimism/pessimism, flexibility, daily computer, phone and cellular phone usage, social support, reflection on health improvement.
*Work-related factors, demand/control, effort/reward*	Work satisfaction, efficiency, competence (sufficiency, development, usage), meaningful work, work atmosphere, work intensity, number of breaks during a regular working day, average working hours and distribution over the week (actual and desired), flexibility of work, general mood on the way to work (sad – happy), working effort, work reward, influence on work situation, work stress, work confidence, support from managers, collegial support, work-place goal clarity and realism, work-place efficiency, reflection on efficiency improvement, priority between health and achievement, time perspectives on decisions at work, existence of serious considerations to quit job, number of sick-leave days, health-economic aspects.

### Blood sampling

The complete list of biological markers analyzed in the current study is presented in Table 3. More biological markers of general nature, such as blood status, were collected for overall health matters or all-purpose profiling. These markers were not analyzed in the present study. Furthermore, P-substance P, S-IL-1beta and P-endothelin were also collected. However, in the first measurement there was not enough blood collected to render the exact results needed for more sensitive analyses of these variables, resulting in the decision to not include them in the present study. Thus, the biological markers analyzed in the present study were only the ones that could be related to various stress-related hypotheses.

**Table 3:** Blood sampling and physiological measures. The table illustrates the biological markers and physiological measures sampled at baseline and after the six months intervention.

**Categories**	**Physiological marker**
*Cardiovascular system and lifestyle*	Blood pressure, pulse, waist-hip ratio, BMI, P-BNP (brain natriuretic peptide), P-PAI-1 (plasminogen activator inhibitor 1), S-insulin, B-HbA1C, S-triglycerides, S-cholesterol, S-HDL, S-LDL, P-fibrinogen, B-trombocytes.
*Stress-related (HPA-axis, catabolic)*	S-prolactin, P-ACTH (adreno corticotropic hormone), S-cortisol, S-TSH (thyroid stimulating hormone), S-T3, S-T4 (free), S-urate.
*Recovery-related (anabolic)*	S-GH (growth hormone), S-IGF-1, S-DHEAS-S (dehydroepiandosterone sulphate), S-estradiol, S-testosterone, S-SHBG (sexual hormone binding globulin).
*Immune markers and neuropeptides*	S-TNFα (tumour necrosis factor alpha), high sensitive S-CRP (c-reactive protein), P-NPY, P-CgA (chromogranin A).

Blood samples were collected from study participants between 7.00–11.30 am at each specific worksite (or nearby). Unfortunately, it was impossible for practical reasons to sample the blood within more narrow time limits. Questionnaires were filled out during the same time period (usually same day or week) in order for the outcome of the blood and questionnaire data to be as comparable as possible. The exact time for blood sampling was recorded for each participant at baseline and at the end of the study so that the blood could be collected at the same time (± 15 minutes). Participants were instructed not to eat or drink (except water), nor use nicotinic substances at least ten hours before blood sampling. The blood samples were analyzed by the Karolinska University Hospital laboratory that is qualified by SWEDAC (Swedish Board for Accreditation and Conformity Assessment) that accredits laboratories in the medical sector according to the standard ISO/IEC 17025. Intra-assay and inter-assay coefficients of variation can be obtained from the laboratory peter.matha@karolinska.se) or by e-mailing the authors dan.hasson@pubcare.uu.se.

### Statistical analyses

The program SPSS 11.5 for windows was used for statistical analyses and an intention-to-treat approach was utilized. This means that all subjects in both groups were included in the follow-up regardless of how much they participated in the intervention programs. And the evaluation is based upon the assumption that everybody – even those who did not participate at all – in the intervention group were compared with everybody in the control group.

Initially, all variables were assessed for normality using Kolmogorov-Smirnov test. Changes over time (time, group and group × time) were assessed using two-way analysis of covariance (ANCOVA). ANCOVA adjusts for initial differences so that the results more precisely reflect possible intervention effects, and thus permits a more sensitive analysis compared to regular analysis of variance (ANOVA). The increase in sensitivity arises from the fact that the covariance reduces the error term (within-group variability) against which intervention effects are compared. Furthermore, ANCOVA is not very sensitive to small deviations from a normal distribution [26]. In the present study, baseline values of the assessed variables were used as covariates. Analyses were in some cases, such as sex hormones stratified with a break up by gender. Finally, to adjust all results of the ANCOVA analyses for the possible effect of multiple comparisons (mass-significance), Bonferroni correction was utilized for each analysis.

Since VAS can be treated as an interval or ordinal scale [27-29] and all variables were not normally distributed, both parametric and non-parametric tests were used where statistically significant differences between the groups were detected in the ANCOVA. Thus, it was decided that changes over time and differences between the groups would only be considered in cases where both parametric and non-parametric tests unanimously were statistically significant. For the non-parametric analyses, new variables (so called Δ variables) based on change between the first and second measurements were constructed. Differences between the groups were then assessed using a Mann-Whitney U test.

Logistic regression was used to model the probability of improvement in the significant Δ variables (dependent variables). Factors such as socioeconomic status, marital status and gender are known to be associated with outcomes in health and stress and were therefore included as covariates in the first step of the regression analysis. Also group was included as a factor in the first step to adjust for possible study group effects. The dependent and independent VAS variables used in the logistic regression were divided by quartile split into high (top quartile) and low (remaining quartiles) categories. Similarly the number of logins was dichotomized by quartile split. The independent VAS variables were selected for two subsequent steps in the regression analysis. The rationale for the second step was to adjust for work related factors that might disturb the relationships, i.e. working hours per week, working atmosphere, work intensity and number of breaks during a working day. The third step included all the remaining dependent variables. In order for the physiological markers to render comparable odds ratio they were dichotomized by quartile split. The fourth and final step included the number of logins to adjust for possible effects of high vs. low frequency of logins to the website.

The specific hypothesis tested in the present study was that the intervention group would improve compared to reference group on biological stress markers and health- and recovery-related ratings captured by the questionnaire. Decreased levels in indicators of catabolism and increased levels in indicators of anabolism were expected in the intervention group compared to the reference group. Since both groups received an intervention, some beneficial changes in the reference group, e.g. in SRH, might be expected as well.

The ethics committees of Uppsala University (Dnr 01–188) and Karolinska Institute (Dnr 01355) approved the research project. A modified version of the web-based tool used in the present study can be found at .

### Role of the funding source

The funding source had no involvement in the study design; in the collection, analysis, and interpretation of data; in the writing of the report; and in the decision to submit the paper for publication.

## Results

**Figure 2:**
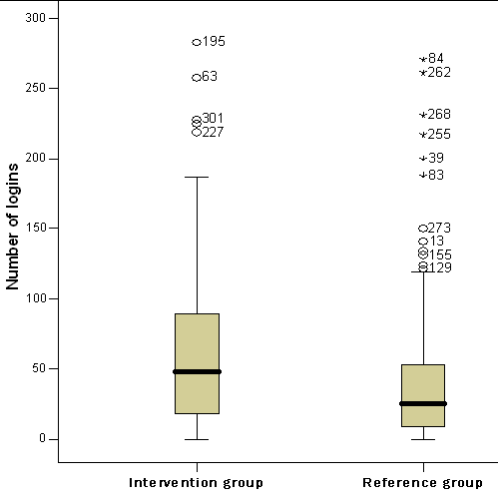
**Website login frequency. **This figure illustrates the number of logins on the website made by the intervention group (median 48 logins) and reference group (median 26 logins) respectively, during the study period of six months (p < .0001, 2-tailed).

Baseline socioeconomic characteristics of the study participants are described in Table 4. Exposure to the interventions, i.e. number of logins to the website, revealed that the intervention group used the website statistically significantly more compared to the reference group (Figure 2; t-test p < .001, 2-tailed; Mann-Whitney U test p < .0001). For the whole sample, the frequency of logins for the lowest quartile was < 10, median 36 and the top quartile >71 logins.

**Table 4:** Baseline socioeconomic characteristics. The table depicts the socioeconomic characteristics age, sex, education, annual income and marital status of the participants (n = 303) from the enrolling IT and media companies.

**Characteristic**	**Intervention group *n *= *129***	***%***	**Reference group *n *= *174***	***%***
***Age***				
≤ 30	31	24	46	27
31–45	44	34	72	41
≥ 46	54	42	56	32

***Sex***				
Male	75	58	112	64
Female	54	42	62	36

Education*				
Compulsory school/High school	54	42	89	51
Academic degree	73	57	83	48

Annual income*				
*< 25,000 USD*	24	18	39	22
25,000 – 40,000 USD	76	59	106	61
> 40,000 USD	27	21	27	16

Marital status*				
Married/co-inhabiting/liveapart	102	79	134	77
Single	25	19	38	22

* 4 missing values (two in intervention and reference group respectively).

**Figure 3:**
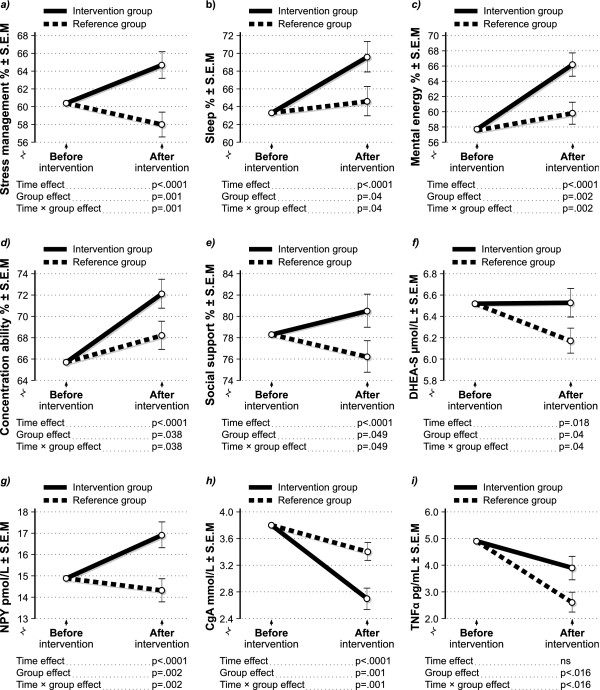
**a-i Two-way ANCOVAs. **The figures illustrate the results of the two-way ANCOVA on the significant outcome measures: a) Stress management ability, b) Sleep quality, c) Mental energy, d) Concentration ability, e) Social support, f) DHEA-S, g) NPY, h) CgA and i) TNFα. All measures are covariated for their own baseline levels.

At the end of the 6-month intervention period, the intervention group had improved significantly as compared to the reference group on ratings of perceived ability to manage stress, sleep quality, mental energy, concentration ability, social support and competence usage at work (2-way ANCOVA, p < .05 time × group effect). With the exception for competence usage at work, all these changes and differences between the groups remained significant when applying the non-parametric Mann-Whitney U test (p < .05, two-tailed). Figures 3a–i illustrate changes in self-rated measures and biological markers over time between the intervention and reference groups, respectively. Results shown are covariated for baseline scores of the depicted outcome variable. SRH increased significantly in both groups, with no differences between the groups (2-way ANCOVA, p < .0001 time effect; time × group effect non-significant). The results of the gender stratified variables were not different from the ones obtained when analyzing the non-stratified data.

Concerning the analyzed blood samples, the levels of the sulphated metabolite of the hormone dehydroepiandosterone (DHEA-S) decreased significantly in the reference group, with no changes in the intervention group. The levels of Neuropeptide Y (NPY) increased significantly in the intervention group compared to the reference group. CgA (chromogranin A) and ACTH (adrenocorticotropic hormone) decreased significantly in the intervention group as compared to the reference group. The levels of the immune marker TNFα decreased significantly in the reference group as compared to the intervention group (2-way ANCOVA, p < .05 time × group effect). With exception for ACTH, all these changes in biological markers and differences between the groups remained significant when applying the non-parametric Mann-Whitney U test (p < .05, two-tailed).

Tables 5 and 6 depict the results of the logistic regression analyses, which were utilized to predict the quartile exhibiting most improvement or beneficial change. The regression models correctly predicted 72.5–80.3% of changes of the various outcome measures. Improvement or beneficial changes in stress management (OR 2.364, 95% CI 1.220–4.578), mental energy (OR 2.194, 95% CI 1.107–4.346), social support (OR 2.752, 95% CI 1.432–5.287), NPY (OR 1.934, 95% CI 1.032–3.623) and TNFα (OR 3.185, 95% CI 1.637–6.196) were significantly predicted in the intervention group compared to the reference group. Thus, the intervention group was approximately two to three times more likely to exhibit the highest improvement quartile in stress management, mental energy, social support, NPY and TNFα. These predictions remained significant even after adjustment for age, gender, annual income, education, marital status, and work related factors that might disturb the relationships, i.e. working hours per week, working atmosphere, work intensity and number of breaks during a working day and all the remaining dependent variables. Beneficial changes in sleep quality, concentration ability, DHEA-S and CgA were not significantly predicted by group in the logistic regression analysis. The frequency of logins to the web was not a significant predictor of changes in any of the dependent variables.

**Table 5:** Logistic regression analyses of self-ratings. The table illustrates the final regression models predicting changes (Δ) in stress management ability, sleep quality, mental energy, concentration ability and social support.

**Predictors of Δ Stress management ability**	**OR**	**95,0% CI for OR Lower – Upper**	**Predictors of Δ Sleep Quality**	**OR**	**95,0% CI for OR Lower – Upper**
Age^b ^	1.041	.655 – 1.654	Age^b ^	.718	.457 – 1,126
Gender^b ^	1.144	.580 – 2.259	Gender^b ^	1.391	.717 – 2.702
Marital status^b ^	1.255	.561 – 2.808	Marital status^b ^	1.175	.536 – 2.572
Educational level^b ^	1.261	.645 – 2.462	Educational level^b ^	.826	.428 – 1.596
Annual income^b ^	1.363	.766 – 2.424	Annual income^b ^	1.532	.856 – 2.740
Group^a, b ^	2.364	1.220 – 4.578	Group^a, b ^	1.638	.854 – 3.141
Δ Concentration ability^f ^	2.754	1.389 – 5.461	Δ Mental energy^e ^	2.343	1.151 – 4.766
Δ CgA	2.563	1.223 – 5.256	Δ Concentration ability^f ^	2.259	1.125 – 4.533
Constant	.002		Constant	.012	

**Predictors of Δ Mental energy**	**OR**	**95,0% CI for OR Lower – Upper**	**Predictors of Δ Concentration ability**	**OR**	**95,0% CI for OR Lower – Upper**

Age^b ^	1.152	.713 – 1,863	Age^b ^	1.348	.844 – 2.152
Gender^b ^	1.319	.659 – 2.638	Gender^b ^	1.250	.633 – 2.469
Marital status^b ^	.998	.429 – 2.320	Marital status^b ^	1.280	.569 – 2.877
Educational level^b ^	.861	.430 – 1.722	Educational level^b ^	1.033	.529 – 2.018
Annual income^b ^	.858	.471 – 1.562	Annual income^b ^	.784	.434 – 1.417
Group^a.b ^	2.194	1.107 – 4.346	Group^a, b ^	.900	.454 – 1.783
Δ Sleep quality^d ^	2.350	1.156 – 4.775	Δ Sleep quality^d ^	2.145	1.052 – 4.376
Δ Concentration ability^f ^	3.831	1.910 – 7.683	Δ Mental energy^e ^	3.638	1.791 – 7.390
Constant	.005		Δ Stress management^c ^	2.171	1.061 – 4.442
			Constant	.005	

**Predictors of Δ Social support**	**OR**	**95,0% CI for OR Lower – Upper**			

Age^b ^	1.456	.920 – 2.304			
Gender^b ^	1.428	.734 – 2.779			
Marital status^b ^	1.822	.836 – 3.970			
Educational level^b ^	.647	.335 – 1.248			
Annual income^b ^	.978	.557 – 1.717			
Group^a.b ^	2.752	1.432 – 5.287			
Constant	.019				

^a ^Reference group: code 1, intervention group: code 2.

^b ^Baseline values.

^c ^Change in stress management ability. Can you manage your stress in general? (VAS, Not at all – Very well).

^d ^Change in sleep quality. How is your quality of sleep in general? (VAS, Very poor – Very good).

^e ^Change in mental energy. How is your energy level in general? (VAS, Empty on energy – Full of energy).

^f ^Change in concentration ability. How is your concentration ability in general? (VAS, Very poor – Very good).

**Table 6:** Logistic regression analyses of biological markers. The table illustrates the final regression models predicting changes (Δ) in the biological markers DHEA-S, NPY, CgA and TNFα.

**Predictors of Δ DHEA**	**OR**	**95,0% CI for OR Lower – Upper**	**Predictors of Δ NPY**	**OR**	**95,0% CI for OR Lower – Upper**
Age^b ^	.863	.547 – 1.363	Age^b ^	.723	.466 – 1.121
Gender^b ^	.527	261 – 1.066	Gender^b ^	.533	.273 – 1.042
Marital status^b ^	1.982	.919 – 4.275	Marital status^b ^	1.510	.710 – 3.215
Educational level^b ^	1.368	.700 – 2.672	Educational level^b ^	1.294	.685 – 2.442
Annual income^b ^	.740	.415 – 1.321	Annual income^b ^	1.404	.807 – 2.442
Group^a, b ^	1.409	.726 – 2.735	Group^a.b ^	1.934	1.032 – 3.623
Constant	.277		Constant	.136	

**Predictors of Δ CgA**	**OR**	**95,0% CI for OR Lower – Upper**	**Predictors of Δ TNFα**	**OR**	**95,0% CI for OR Lower – Upper**

Age^b ^	.643	.411 – 1.006	Age^b ^	.915	.593 – 1.412
Gender^b ^	1.449	.749 – 2.803	Gender^b ^	1.357	.698 – 2.638
Marital status^b ^	.767	.339 – 1.737	Marital status^b ^	.458	.182 – 1.154
Educational level^b ^	.732	.380 – 1.409	Educational level^b ^	1.508	.778 – 2.925
Annual income^b ^	.891	.502 – 1.581	Annual income^b ^	1.228	.695 – 2.169
Group^a, b ^	.629	.324 – 1.221	Group^a.b ^	3.185	1.637 – 6.196
Δ Stress management^c ^	2.343	1.157 – 4.744	Δ Stress management^c ^	.404	.182 – .895
Constant	.887		Constant	.130	

^a ^Reference group: code 1, intervention group: code 2.

^b ^Baseline values.

^c ^Change in stress management ability. Can you manage your stress in general? (VAS, Not at all – Very well).

## Discussion

In the present study we evaluated whether or not a web-based tool, designed for health promotion and stress management, reduces stress and increases physiological markers and psychological ratings of health, recovery and general well-being. At the end of the 6-month intervention period, the intervention group had improved significantly as compared to the reference group on self-ratings of perceived ability to manage stress, sleep quality, mental energy, concentration ability and social support. SRH increased significantly in both groups, with no differences between the groups.

### Questionnaire

A striking finding is that that ratings of sleep quality improve in the intervention group vs. reference group together with related systematic findings in biological markers and other self-ratings. There is emerging evidence suggesting that sleep alterations can modulate the stress-health relationship. Acute and chronic stressors are associated with subjective and objective measures of sleep disturbances [30]. Thus, improvements in sleep quality might mediate some of the stress protective and health promoting effects found in the intervention group.

Since all participants received some kind of intervention, some beneficial changes were expected in both groups. As a matter of fact, there were several health-related statistically significant improvements for both groups over time (time effect). To mention some, ratings of SRH, eating habits, memory, physical activity, self-esteem and work joy improved as well as levels of cortisol and cholesterol that decreased. However, as the groups did not differ (time × group effect was ns) it is not certain that these effects can be attributed to the web-based tool although they might have resulted from it. To draw such a conclusion a third, passive reference group would have been needed, which was not possible for budget reasons.

The findings of the present study are in line with previous computer-based intervention studies with cognitive exercises that have shown beneficial effects on affective states, such as depression and anxiety [11-13], stress management [14] and insomnia [16]. Results of the present study are further confirmed in a prospective non-controlled study, in which a web-based intervention was found to decrease ratings of loneliness and depression, whereas perceived social support and self-esteem increased [31].

### Biological markers

In the present study, DHEA-S decreased significantly in the reference group but remained unchanged in the intervention group. DHEA-S is a steroid hormone that has anabolic as well as neuroprotective effects. DHEA-S has also been found to counteract the effects of corticosteroids, such as cortisol, and to be inversely related to both stress and cortisol [32,33]. Thus, the DHEA-S decrease in the reference group may indeed be a consequence of physiological stress caused by the turbulence that occurred in connection with the study period. This indicates that the intervention program might be protective against stress and facilitate recovery, since DHEA-S remained unaltered in the same stressful period in the intervention group. Furthermore, a number of studies have suggested that DHEA-S can have beneficial effects on cognition, metabolism, wellbeing, and vascular and immune function [32-34]. Considering such prior knowledge, it is of interest that we in the present study found concurrent improvements in DHEA-S and a range of cognitive functions, such as improved concentration ability and increased mental energy in the intervention group.

NPY increased significantly in the intervention group as compared to the reference group. NPY is a hormone that has been reported to have a soothing, anxiolytic as well as antidepressive effect in the central nervous system [35]. The anxiolytic effects of NPY are probably mediated by Y1 receptors in the amygdala and involve inhibition of corticotrophin-releasing hormone (CRH). Moreover, NPY inhibits hypothalamus-pituitary-adrenal (HPA) activity and is thereby effective in reducing secretion of CRH, adrenocorticotropic hormone (ACTH) and cortisol. Finally, NPY has been found to promote and improve sleep [35]. Consequently, the increase in NPY found in the present study may partly explain the beneficial effects, including sleep improvement, found in the intervention group. Some of the findings of the previous literature however, are hard to apply to the present study since they are based upon pharmacological doses of NPY.

Chromogranin A (CgA) decreased in both groups, but significantly more in the intervention group as compared to the reference group. CgA is stored in the core of catecholamine vesicles and is often, but not always co-released with catecholamines. Secretion occurs only during marked activation of the sympathochromaffin system and only stimuli strong enough to induce catecholamine secretion are associated with CgA release. However, CgA also shows ultradian variation, which does not appear to be linked to modifications of catecholamine release [36,37]. It has been suggested that in situations of mild mental stress CgA is stable and slow to respond [38]. The decrease in CgA in the intervention group might indicate a lesser activation of the HPA-axis and a higher activation in the reference group, perhaps combined with a reduction in activity related to seasonal variation. Cortisol production, for instance, usually declines during the autumn. This might explain why CgA decreased more in the intervention group.

One of the major inflammatory cytokines, TNFα, decreased significantly in the reference group compared to the intervention group. TNFα is one of many markers of the immune system, and the production increases during immunological, inflammatory and stress responses [39]. It has been suggested that cytokines are involved in the regulation of HPA-axis activity [40]. For example, TNFα increases the secretion of CRH (corticotrophin releasing hormone) from the hypothalamus, which in turn results in an increased secretion of ACTH. In turn, ACTH stimulates the release of glucocorticoids from the adrenal cortex. During chronic stress, however, there seems to be a poor relationship between ACTH plasma concentrations and the release of glucocorticoids [39,41]. Thus, at present there is insufficient information concerning the relative effects of acute and chronic stressors on cytokine activity [42]. It has however been shown that TNFα as well as other hormones, such as cortisol and DHEA-S, exhibit seasonal variation in production. The production of these biological markers seems to be elevated in the spring time and reduced during the autumn [19,43]. This seasonal variation, i.e. reduction in the autumn, in combination with long-term stress might explain why TNFα decreases in the reference group compared to the intervention group. This possibly stress-related reduction might partly have been counteracted in the intervention group that showed improved self-rated as well as physiological stress management abilities. Consequently, since DHEA-S also remained stable in the intervention group as compared to the reference group that decreased this might be regarded as a systematic finding indicating better stress management and/or decreased stress level in the intervention group as compared to the reference group.

### Methodological considerations

Everything was completely web-based from the start in the present study. It means that the stress management tool was utilized and assessed via the same medium that was used for collecting self-ratings and other relevant background data. This automated, interactive self-help approach differs from previous studies of web-based interventions. Most commonly, other studies have been more similar with face-to-face counseling, where in addition to a website an active counterpart, often a psychologist, issues assignments and evaluations via e-mail. Consequently, the results of the present study might not be completely comparable with other assessments of web-based intervention studies.

The intervention and reference group were treated in the same way concerning blood sampling, advice, web-based questionnaires, etc. The only thing that distinguished the groups was the addition of the interactive cognitive exercises and a chat for the intervention group, which indicate that the complete web-based health promotion and stress management system contributed to the beneficial effects on health, well-being and recovery.

A multitude of items (57) and physiological markers (30) were analyzed in the present study, which makes it relevant to discuss the possible problem of mass-significance. To clarify this issue, percentages of significant findings out of the total number of analyses are presented. Altogether 87 parametric analyses were conducted on relevant VAS items and physiological markers, out of which 11 (13%) significant results were obtained and 9 (10%) remained significant when non-parametric tests were used. Additionally, for several of the biological markers there may be systematic variations in levels during the sampling interval (7.15–11.30 am). For instance, serum cortisol may start decreasing. Most of this variation takes place between morning and evening however. The circadian variation during the morning hours may introduce additional random error in our results. Since there was no systematic difference in sampling hours between the two groups, no systematic bias is likely to have arisen due to this source of error.

The study period of six months might not be enough to cover long-term effects. Accordingly, the beneficial effects found in the intervention group compared to the reference group might be attenuated or continue to improve on a longer term perspective. Therefore, a post intervention follow-up was conducted six months after termination of the study, i.e. twelve months after initiation of the study. The result of this post intervention long-term follow-up will be presented in a future article. However, there are indications that some of the beneficial improvements found in the present study are attenuated after 12 months. Another aspect is that an intervention that focuses solely on individuals might have less ability to produce a lasting effect compared to interventions that also consider organizational aspects. Such multidimensional interventions could perhaps increase the possibilities for the participants to pursue beneficial effects of the individually focused intervention.

Finally, it was mentioned in the introduction that the study was conducted during a high stress period. Therefore, the general health status and occurrence of stress-related problems among the study participants might be discussed. However, apparently the participants were healthy enough to be at work and at baseline there were no participants on sick leave. Furthermore, in the case of participants going on sick-leave they could register these changes in the "profile" section on the website.

There were some weaknesses with this study, e.g. incorrect e-mail addresses to some participants complicated or made communication impossible. Furthermore, we have no exact number of potential participants in the study. This fact might bias the results considering that the sample might not be representative for all the employees. However, in general there was a great interest among the employees of the enrolling departments to participate, as for instance in one of the departments where we have the total number of potential participants, 95% enrolled. Similar participation rates are therefore estimated for the other departments. In any case, based on approximation of the total potential number of employees at each department, enrollment rate was most probably not less than 80% in the worst case scenario.

### Implications and future directions

The results of the present study imply some short-term beneficial effects from a web-based tool for stress management. However, initial analyses from a long-term post intervention follow-up indicate a reversion for some of these beneficial effects. Future studies would benefit from pilot testing the web-based tools and thereto related functions to reduce risks of computer-based malfunctioning. Furthermore, logging of usage patterns may contribute with knowledge about how web-based interventions could be improved. More studies assessing psychological as well as physiological effects are needed before more firm conclusions could be drawn.

## Conclusion

In summary, the current study suggest that an automated web-based system for self-assessments and real-time feedback of scorings, combined with cognitive exercises, might be beneficial to counteract unwanted stress and improve mental and physiological indicators of health at least during a six-month intervention period. Thus, such a web-based system may be an opportunity in counteracting some clinically relevant and common stress and health issues of today.

## List of abbreviations

ACTH Adrenocorticotropic hormone

ANCOVA Covariated analysis of variance

ANOVA Analysis of variance

CgA Chromogranin A

CRH Corticotropin releasing hormone

DHEA Dehydroepiandosterone

HPA Hypothalamus-pituitary-adrenal

IT Information technology

NPY Neuropeptide Y

NS Non significant

REM Rapid eye movement

SD Standard deviation

S.E.M Standard error of the mean

SRH Self-rated health

TNFα Tumour necrosis factor α

VAS Visual analogue scale

## Competing interests

Following the termination of this study, BA and DH have commercialized the web-based health promotion and stress management tool.

## Authors' contributions

BA and DH have designed the study and compiled the questionnaire. DH was responsible for overall project management. DH conducted the statistical analyses and drafted the manuscript, with substantial and essential input from TT, UMA and BA. All authors have read and approved the final version of the manuscript.

## Pre-publication history

The pre-publication history for this paper can be accessed here:


